# A SHARED study-the benefits and costs of setting up a health research study involving lay co-researchers and how we overcame the challenges

**DOI:** 10.1186/s40900-016-0021-3

**Published:** 2016-03-03

**Authors:** Carole Mockford, Matt Murray, Kate Seers, Jan Oyebode, Richard Grant, Sue Boex, Sophie Staniszewska, Yvonne Diment, Jim Leach, Uma Sharma, Rosemary Clarke, Rashida Suleman

**Affiliations:** 1 Royal College of Nursing Research Institute, Division of Health Sciences, Warwick Medical School, University of Warwick, Coventry, CV4 7AL UK; 2 Alzheimer’s Society, London, UK; 3 School of Dementia Studies, University of Bradford, Bradford, UK; 4 University/Users Teaching and Research Action Partnership (UNTRAP), University of Warwick, Coventry, UK; 5 Alzheimer’s Society Research Network, London, UK; 6 Patient and Public Action Group, Comprehensive Local Research Network, Coventry, UK

**Keywords:** Lay co-researchers, Service users, Health research, Payment setting, NHS Trust approval, Dementia, Memory loss

## Abstract

**Plain English summary:**

In the United Kingdom (UK), official bodies such as the Department of Health and research funders such as the National Institute for Health Research support and encourage lay involvement in all stages of research studies. The SHARED study has had substantial patient and public involvement (PPI) from developing the idea to dissemination. The aim of the study has been to develop recommendations led by service users for health and social care professionals to use at hospital discharge and in care planning for people living with memory loss and their carers. This article is about how the study started and the benefits, costs and challenges we encountered as the lead and lay co-researchers. Once we were successful with the grant application, we had to recruit and train the lay co-researchers and obtain various approvals before we could start the project. We had various support from funders, the Research Ethics Committee, lay members of Alzheimer’s Society and from the lay co-researchers. However, we encountered some challenges with paying the lay co-researchers and with getting the approval for the co-researchers to interview staff on NHS premises. The challenges were overcome eventually but some aspects of the study changed because of this. We suggest that some changes could be made to the research system which would lead to greater inclusion of the lay co-researchers in research studies and would make the process more straightforward for the research team.

**Abstract:**

**Background**

Involving patients and the public in all stages of research has been the focus of the SHARED study. Patient and public involvement (PPI) is an important strategic priority for the Department of Health and funders such as the National Institute for Health Research. The aim of this paper is to describe the benefits, challenges and costs involved in setting up the research study with lay members as part of the research team. The study focused on developing service user-led recommendations for people with memory loss and their carers, on discharge from acute hospital to the community.

**Methods**

This began with a discussion of an initial research idea with a lay group of carers and people living with dementia. Once funded, approval was sought from the Research Ethics Committee and NHS Trusts to conduct the research including the active involvement of lay co-researchers. Finally, to recruit, train and pay lay co-researchers in their role.

**Results**

The benefits of PPI have included developing ideas which are important to people living with memory loss; support for PPI received from the funders and research ethics committee, high levels of interest from volunteer groups, and lasting enthusiasm from many of the co-researchers. Organisational challenges were met in the requirement for research passports and with payment methods for the co-researchers. Training was beneficial but incurred extra costs for repeated training days.

**Discussion**

Overall the benefits outweighed the challenges which were overcome to varying degrees. The lay co-researchers gained membership of a study group and a beneficial partnership developed with the third sector. The biggest challenge was in overcoming the differences in approach to lay co-researchers between NHS Trusts. Organisational culture has been slow to incorporate PPI and this has not yet been fully addressed. It has the potential to delay the start of projects, affect recruitment time, incur extra research costs and disadvantage PPI.

**Conclusion**

Buy-in to service user involvement in research studies could be improved by clarifying the requirements for NHS Trust approval and by simplifying the system for financial reimbursement to lay co-researchers. This would improve inclusivity and provide a smoother process for the research team and the co-researchers.

## Background

In 2006, the Department of Health in England set the goal of involving patients and the public in all stages of the research process [1]. This became an important strategic priority for a major funding body, the National Institute for Health Research (NIHR). The NIHR funding body strongly supports the inclusion of patient and public involvement (PPI) in research reflecting trends in the United Kingdom and internationally [2, 3]. It also funds INVOLVE, an organisation supporting public involvement in the UK National Health Service (NHS) public health and social care research [4]. A recent review of public involvement in the National Institute for Health, Going the Extra Mile [5] has celebrated the value and achievements of public involvement in NIHR funded research and has set an agenda for moving forward.

Recent studies on the involvement of service users in health research have shown that benefits include: more informed communities, more relevant research topics, creating links with seldom heard groups and developing better targeted resources [6]. Increasing numbers of research studies are involving service users in health research such as co-applicants on grant applications, as members of advisory or steering groups and increasingly as lay co-researchers. Whilst Patient and Public Involvement (PPI) is beginning to embed itself in research culture and more literature is becoming available on how this impacts on research findings, [6] building PPI into an organisational culture is more difficult [7]. It is less common for studies to include lay co-researchers, and there is little information on the process itself particularly the benefits, challenges and costs arising from this. This article describes the experiences of the lead researcher, the lay co-researchers and a supporting charity in the setting up of the Services after Hospital: Action to develop Recommendations (SHARED) research study. (The SHARED study findings will be reported elsewhere). England is leading the world by example in PPI in research [8] but there are still some barriers to the change of organisational culture. This article describes how some of these challenges were overcome and how some aspects of the study were changed as a result.

## Aim

The aim of this article is to report the benefits, challenges and costs of setting up a health research study with lay co-researchers.

The aim of the SHARED study has been to develop service user-led recommendations around discharge from acute hospital care to community care for people living with undiagnosed memory problems or dementia and their carers. The objectives were to explore the experiences of patients and carers of health and social care services post discharge; to explore the extent to which patients and carers were involved in the discharge process and to examine the experiences of health and social care professionals.

REC approval: NRES committee London: Camberwell St Giles 14 LO 05/01.

## Methods

The study idea was initially discussed with the Alzheimer’s Society Research Network which consists of carers and people living with dementia. Once funding was awarded by the National Institute for Health Research (Research for Patient Benefit), the lead researcher applied for approval from the Research Ethics Committee and three NHS Trusts (sites 1, 2 and 3) to conduct the study which was to include the active involvement of lay co-researchers in the collection of data in two of the NHS Trusts. Semi-structured interviews were to take place in the homes of people living with memory loss and their carers after discharge from an acute hospital, and again at 6 and 12 weeks post discharge. The patients were also to be aged 65 and over, and to have been an in-patient for at least one week. Semi-structured interviews were to take place with health and social care professionals, and Admiral Nurses. The co-researchers would be involved in data analysis and in the facilitation of focus groups of study participants (people living with memory loss and their carers). Recommendations for people living with memory loss and their carers on leaving hospital to return home would be shaped and finalised by study participants at the focus groups.

Arrangements were made to recruit lay co-researchers from the Alzheimer’s Society Research Network, User/University Teaching and Research Action Partnership (UNTRAP) at Warwick Medical School, and the Clinical Local Research Network Patient and Public Action Group (CLRN PPAG).

UNTRAP delivered training sessions on research ethics and general research methods; interviewing techniques and interviewing practical experience; data analysis and facilitating focus groups.

Background checks, indemnity insurance and payment to the co-researchers were arranged via an Alzheimer’s Society study group set up for the SHARED study.

## Results

Below we describe the development of the study idea and the process of setting up the SHARED study with 12 lay co-researchers. We report on some of the costs involved, how we were supported and encouraged by the many benefits we encountered and how we managed to overcome the organisational challenges we faced. The lay co-researchers were asked to maintain a journal throughout the study to document their experiences, some of their thoughts and experiences have been incorporated into this article.

### Benefit - Service users: developing a meaningful research study

**Fig. 1:**
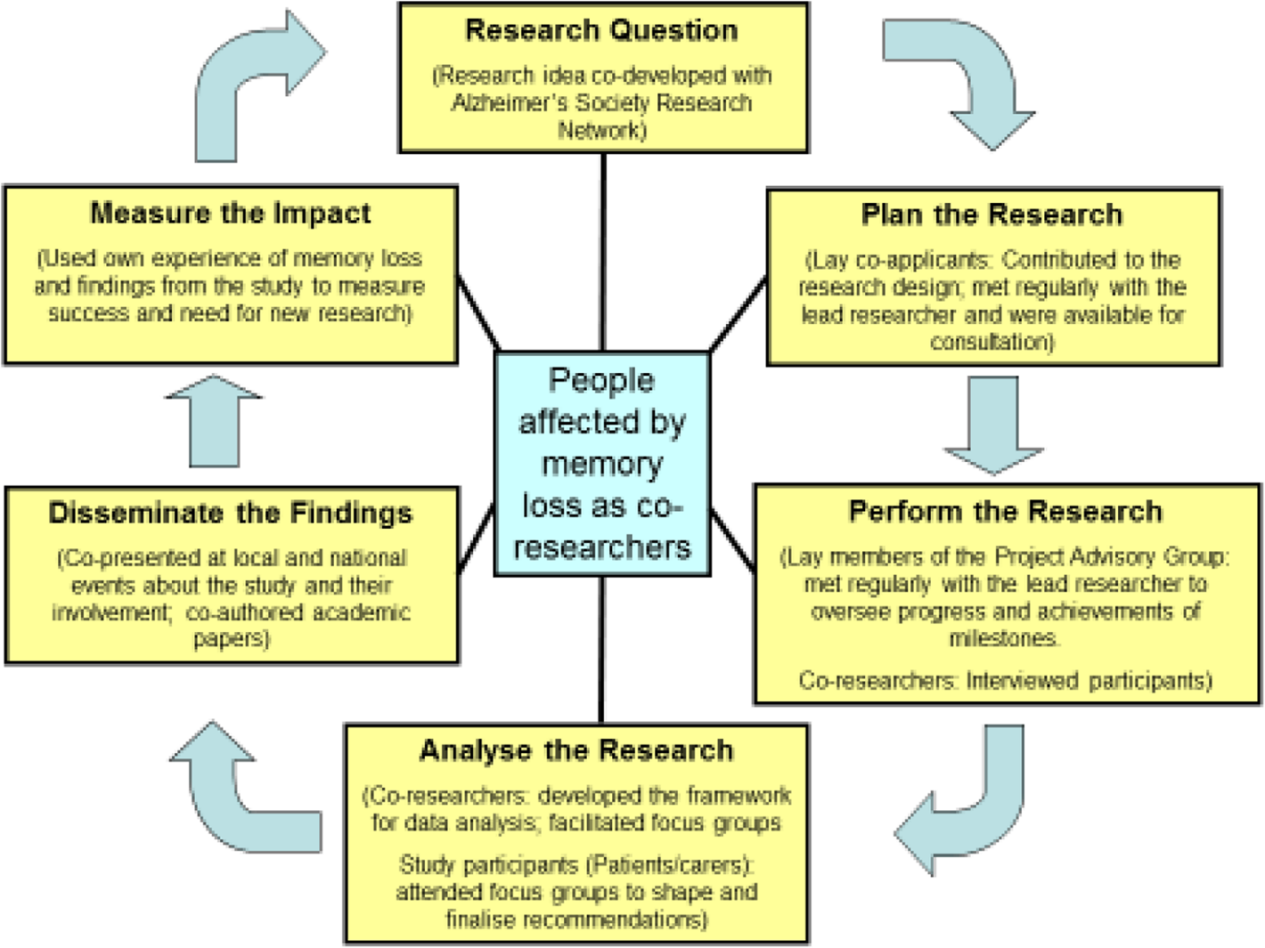
The SHARED research cycle. (adapted from Alzheimer’s Society Research Network)

The SHARED study has involved service users across the research cycle from the development of an idea to the dissemination of the findings (see Fig. 1).

Lay members of the Alzheimer’s Society Research Network, [9] all of whom have personal experience of dementia, were originally consulted in 2011 by the lead researcher as part of an NIHR initiative. An initial idea for a study to develop recommendations for the transition from hospital discharge to formal care (long-term care home) for people with dementia and their carers was discussed. However, it became clear during discussions that returning home with varied or no support was also an important issue which urgently needed to be addressed. Together with the research team which included two lay members, the proposed study was therefore changed to focus upon the transition from an acute hospital back to the community. An initial grant application was unsuccessful but received positive reviews. Further lay reviewer feedback was obtained which positively supported the inclusion of lay researchers and the topic was well received. A suggestion from lay reviewers to increase the sample size of paired patients and carers to allow for ‘drop-out’ was incorporated and this refined the final and successful funding application.

Service users were co-applicants in the Project Team and became co-investigators. They collaborated on the development of the study, met regularly as part of the team and were available for consultation during the course of the study. Service users were also members of the Project Advisory Board who along with health professionals and academics, oversaw the project to review progress and the achievement of milestones. Service users were co-researchers who collected interview data, made a major contribution to the data analysis, facilitated focus groups and disseminated the findings with the lead researcher.

### Benefit – Funders: awarded substantial costs for lay co-researcher time and expenses

Funding included an hourly rate and travel expenses for each co-researcher who participated in the study. The amount paid for research work undertaken by lay co-researchers was decided by offering an equal amount to that offered by the local lay participation group at the University of Warwick Medical School, the User/University Teaching and Research Action Partnership (UNTRAP) of £18 per hour (later increasing to £20 per hour). This also concurred with other examples of payment setting [10]. Costs included the hire of a paid care worker (at £11.20 per hour; the erstwhile rate of a national carer company) if informal carers attended any of the three training days. The funders requested that formal training was organised for the lay co-researchers, rather than that offered by the research team, and further funding was awarded to cover this cost.

### Benefit – The Research Ethics Committee: their support and suggestions

The Research Ethics Committee were very supportive of the inclusivity of the study. At the time we had suggested that current and past carers be invited to be co-researchers but the committee felt that that did not go far enough and they imposed a condition. The Committee recommended participation was extended to people living with undiagnosed memory problems not just people diagnosed with dementia and that the co-researcher role be offered to these groups too, to strengthen the lay involvement.

### Benefit – Volunteer groups: easily accessible lay interest in research

An invitation to participate in a research study was sent to lay members of existing user groups who were interested in research. These groups included the Alzheimer’s Society Research Network (HQ London), the User/University Teaching and Research Action Partnership (UNTRAP) at Warwick Medical School, University of Warwick and the Comprehensive Local Research Network Patient and Public Action Group (CLRN PPAG). Whilst not the ‘general public’, some were volunteers for charitable work, and others offered their personal ‘lay’ expertise for research activities such as committee work. None had experience of being a researcher. UNTRAP was specifically set up to meet the requirements of lay representation to use their existing skills gained from real life experience to best effect. Each group had a different mission and the lay co-researchers provided this study with different perspectives and varied experiences.

The lead researcher sent an email advertisement for 12 volunteers who either had experience of caring for someone with memory loss or dementia, or were people who lived with memory loss or dementia, to work as co-researchers on a research study. We estimated that about a quarter would drop-out or be unavailable for interviews and that approximately nine co-researchers would ultimately be available to conduct three interviews at each of three data collection points, and this would not be too burdensome.

Those interested were given three weeks to respond with a reminder sent one week prior to the deadline.

*‘For me, the prospect of active engagement as a co-researcher put the proposal at a level above that of being on a Management Committee. There was an excitement about the proposal which marked it as a quality opportunity and a worthwhile experience’ (CR03)*

The numbers of responses exceeded the number of co-researchers needed and because of this some volunteers were declined e.g. because their experience did not include memory loss as specified. These people were thanked for their interest and encouraged to maintain their interest for other studies. Of those who did have experience of memory loss, 12 volunteers were accepted onto the study as co-researchers, others were interested in becoming members of the Project Advisory Board. The twelve lay members were from various backgrounds and with varied experience of memory loss or dementia mostly as previous carers or close relatives of someone who had dementia. Only one was a current carer. There were no responses from people living with memory loss or dementia.

*‘I have been involved with the work of UNTRAP for the past many years. My voluntary commitment to this group has been both due to my personal journey of being a family carer at a very young age and also my professional commitment of offering support to family carers and service users’. (CR06)*

### Benefit – Training sessions: skills and confidence were gained by the lay co-researchers

Funding was awarded to include three training sessions provided by UNTRAP at the University of Warwick at a cost of £500 per day. In addition, the cost of a professional carer was offered for carers who needed support at home whilst they were away.

Training co-researchers is a contentious issue by some since a trained co-researcher is considered to lose their lay perspective by becoming more professional than lay [11, 12]. As co-researchers who are conducting sensitive, semi-structured interviews in a vulnerable participant’s home it can be argued that they must receive some degree of training or guidance. The University’s researchers’ code of practice [13, 14] requires that researchers meet legal and ethical requirements for research, and places responsibility on the lead researcher to ensure that study researchers have the necessary training, time and resources to carry out the role. Although there is no precedent at the moment, this arguably must apply to lay co-researchers too. All of the co-researchers in this study were new to the role but some had experience of interviewing in other contexts e.g. completing forms with clients in previous employment but not in a research capacity. Training gave the co-researchers an insight into their own and others’ characters and helped with team building and peer support. It also gave the lead researcher an indication of their ability for undertaking the role.

*‘Shared learning made our journey much more interesting. Professional and personal experiences in the training sessions shared by the individuals, reflected their commitment to this piece of work’. (CR06)*

The co-researchers appeared to gain confidence in what they were expected to do. They did not appear to lose their lay perspective, in fact it increased their confidence to challenge, initially as a group and subsequently as individuals. It also encouraged them to contribute their thoughts and ideas to the research methods and throughout the study.

*‘Training built [our] capacity to more successfully undertake the role, whilst maintaining [our] lay perspective’ (CR03)*

Training sessions were organised whilst the background checks were ongoing. Three one-day sessions were planned which firstly included: research ethics and confidentiality followed by a general overview of research methods; interviewing techniques and interviewing practical experience; and some weeks later, data analysis and conducting focus groups. Each training day was over a six hour period with a half hour break for lunch. All sessions were held at Warwick Medical School.

*‘Feeling a little apprehensive meeting up with [lead researcher] and other volunteers as I have not done this sort of thing before. Soon put at ease and training very good. Interesting day with discussion about consent in the afternoon’. (CR01)*

Eleven of the co-researchers attended one or more sessions. They were awarded a certificate for the sessions they attended. The skills they learned over the course of the study are transferable and have the potential to build capacity for other research studies.

### Overcoming Challenges – Negotiating mechanisms for payment and conducting background checks

It had proved challenging to arrange appropriate checks, payment, and insurance indemnity for the co-researchers via the University. After much discussion and contact with those with various expertise of working with lay co-workers including INVOLVE, [4] it was decided that they would have to be employees which would incur much paperwork and time delays on both sides. Instead, an opportunity was negotiated with Alzheimer’s Society to form a study group for the co-researchers. The advantage was that the co-researchers could register their background checks (Disclosure and Barring Service (DBS) and occupational health checks), and submit their expenses and honorarium claims for payment as part of a specific group. A formal arrangement was set up between the University and Alzheimer’s Society to cover outgoings and the cost of the extra administrative work.

Completed DBS and occupational health forms, together with other documents of identity were taken by each co-researcher to the local Alzheimer’s Society offices for checking. Once checked, documents such as birth certificates or passports were immediately returned to them. However, the process was difficult for some and a few co-researchers found the DBS process stressful as they misunderstood the type of documents needed and this warranted one or more further visits to their local Alzheimer’s Society office.

*‘For some this was a new procedure for them and was more problematic than for those who had been through the process previously. I remember the first time I went through the process it seemed cumbersome and difficult but actually it was quite easy. I am now much more relaxed about the process’. (CR03)*

Delays in the administration of one occupational health check and the loss of one DBS certificate by a local office prior to it being recorded, prevented two co-researchers from interviewing at the beginning of the data collection period although they were eventually able to interview later on.

*‘It took a few weeks for my first DBS clearance to come through. I was quite upset when the paper work was lost. Thankfully I was able to share my concerns with the lead researcher. I was anxious that the other lay researchers had already started the interviews and I was still going through the rigmarole of the DBS second clearance for another few weeks’. (CR06)*

It took between two and eight months to organise the involvement of every co-researcher in this study from the initial advertisement. Most had successfully completed the process within two to four months.

### Overcoming Challenges – maintaining interest and positivity throughout the prolonged administration process

Co-researchers attended training sessions during the lengthy administration process. They were also in email contact with each other and kept up to date with the progress of the study by the lead researcher. Seven of the original 12 people who expressed interest in the role reached the stage of being able to interview participants. Three withdrew from the study partly due to the complications of providing the supporting documentation needed for the enhanced DBS process (although all eventually obtained a DBS response), and two withdrew as they were not able to give their commitment to the study.

### Overcoming Challenges – extra costs incurred for training

Not all participants could attend the planned dates for the first two training sessions to prepare them for interviewing. Six co-researchers attended the first session and nine attended the second. Extra costs for additional training sessions were incurred for co-researchers who were not able to attend on the planned days. To reduce costs some of the repeated training was delivered by the lead researcher.

### Overcoming Challenges - research passports for the co-researchers, conflicting requirements

Whether or not research passports were required for co-researchers became an issue. The purpose of a research passport is to satisfy NHS Trusts’ needs for research governance without undue delay to research projects by providing researchers with a ‘passport’ containing clearance from several bodies which is then transferable between NHS organisations. To establish a passport includes obtaining occupational health and DBS clearance, as well as signatures from line managers, human resources and individual NHS Trusts. The process takes several weeks and it is not clear who should sign the form as line manager or from human resources for a lay person. When applying for NHS Trust approval from each of the NHS sites, two different approaches were encountered. The host NHS Trust for site 1 decided that research passports were not required for the co-researchers as Alzheimer’s Society were providing insurance indemnity. The second NHS Trust for site 2 did request research passports if the co-researchers did not already hold an employment contract with the Trust and they wished to conduct research activities on NHS premises. After long discussions with both NHS Trusts it was clear that the lay researchers would need research passports for interviewing staff at site 2. As recruitment of study participants had already started in site 1, recruitment in site 2 would have been severely delayed due to the extra time needed to administer this. It was therefore agreed that co-researchers would not interview staff at site 2, and only the lead researcher, who had a research passport, would conduct these interviews. A third NHS Trust (site 3) was introduced into the study at a later stage in order to interview Admiral Nurses who worked in the community. In order to obtain approval quickly, the lead researcher undertook to conduct one group interview in site 3 on NHS premises and did not include the co-researchers in the process.

*‘We just wanted to ‘get on and do a good job’. CR03*

In addition, both Trusts for site 1 and site 2 requested ‘wet ink’ signatures on curricula vitae from the co-researchers which caused a slight delay.

### Benefit – the lasting enthusiasm of the lay co-researchers

Despite some of the difficulties in setting up the SHARED study, it was heartening that those who stayed the course were as keen to get started on the data collection as they had been in answering the recruitment call.

*‘E-mail and ‘phone call from [lead researcher]. First participant ready and as I have had checks – looks like it is going to be me! Got to go through the questions again as I seem to have forgotten most things in the summer heat!! Charge ‘phone’. (CR01)*

Two of the seven co-researchers withdrew after data collection due to personal reasons but five continued to the end of the study.

*‘I always felt that my contribution was valued..’ (CR02)*

*‘Throughout the project the co-researchers felt ‘valued’ and that they were adding value to the work. This was of personal benefit to us, particularly those ‘retired’ from regular work’. (CR03)*

## Discussion

Overall the lead researcher felt that the benefits of working with lay co-researchers outweighed the administrative challenges.

There are varied and various groups of enthusiastic people who want to be, and are involved in research studies, usually as members of management committees but less so as co-researchers and the SHARED study has provided the opportunity for building capacity for future studies. Lay input into the SHARED study was substantial, crucial and helped to shape the focus of the research into something valued by people in the community. With the setting up of a study group a beneficial partnership grew with Alzheimer’s Society. The benefits of partnership working with the voluntary sector have included providing a group identity for the co-researchers, a ‘real world’ underpinning for academic research and an important route to disseminating findings to those who matter.

Training provided an opportunity not only to cover the basic skills for being a researcher, it also helped to develop confidence in the co-researchers to raise issues they did not understand or agree with, and it helped them to understand how research is undertaken.

There is a strong message of encouragement from policy and research funding bodies to include a lay perspective in all stages of the research process. The SHARED study has included lay input from the development of the idea to dissemination. There has been financial support from funders, a positive response from the Research Ethics Committee, practical support from the voluntary sector, and lastly a great deal of enthusiasm from the lay members of the study. However, there are places where support for PPI is still in its infancy such as in the administration of research studies and some problems need to be addressed. Challenges were overcome eventually but not always satisfactorily and future studies may need to prepare for other solutions.

We had tried to avoid the situation where individual co-researchers might be burdened with too many interviews. We experienced unexpected cross-over where some co-researchers were ready to start data collecting whilst additional training days for others were taking place and background checks for some were taking a long time, resulting in more opportunities for the early starters to undertake interviews with study participants. The co-researchers were given the opportunity to refuse to interview particularly if they felt overburdened, however, no-one suggested this was the case.

The two key challenges to the setting up of this study were firstly how to pay the co-researchers: as employees or as members of a study group, and secondly, whether or not they should go through the professional researcher route of having to complete a full research passport to satisfy some NHS Trusts’ guidelines, particularly where a third party holds the insurance for the co-researchers. These challenges were overcome but changed some aspects of the study. The lay co-researchers who were recruited from UNTRAP and the CLRN PPAG had little choice but to become members of Alzheimer’s Society Research Network, which is a system matured in administering the work of volunteers. However, a strong partnership grew from this connection which has been beneficial for publicising the study and later disseminating the findings. Secondly, the co-researchers were going to interview health and social care professionals in both NHS Trusts, but due to the need to have a research passport in one Trust they had less opportunity to do this. The requirement for research passports by NHS Trusts needs to be clarified and time allowed for this lengthy procedure. Guidance for gaining Trust approval for lay co-researchers is currently unclear [15]. If research passports are required for lay co-researchers, who are accompanied by a lead researcher with a research passport, then a system of having a line manager and a link to Human Resources also needs to be put in place for them. The key disadvantage of providing a line manager, a link to Human Resources, and being paid as an ‘employee’ places the lay co-researchers in the position of losing their ‘lay’ identity as they become more affiliated to an employer. Although there may be arguments to support this, there needs to be a conversation around the ongoing discussion of ‘how lay is lay?’.

This article suggests that whilst there have been many benefits to involving service users in this study, some organisational aspects have been difficult to negotiate particularly the mechanisms for payment and obtaining NHS Trust approval.

It was difficult to foresee how budgetary estimates would work over the course of the study and it was dependent on how many co-researchers stayed active to the end of the study. Extra costs were later offset by discounted places for lay co-researchers at national conferences.

For the most part, the lay co-researchers were unaware of the setting-up difficulties and feedback suggests that the experience of becoming a lay co-researcher was a positive one.

## Conclusion

Research funding bodies, exemplified by the NIHR who fully support patient and public involvement in research, may need to go further than the ‘extra mile’ [5] to address the issues found in setting up the SHARED study. Researchers need the full support of the Research and Governance infrastructure to help include lay co-researchers in a study, to maintain interest to participate and to avoid long delays to the start of the study. A discussion is needed around the requirement for research passports for lay co-researchers and in what circumstances they are needed by some NHS Trusts. The organisational barriers we experienced were overcome but need to be addressed for future studies. This can potentially delay the start of the project, affect recruitment times, incur extra research costs and disadvantage PPI. This is particularly pertinent as PPI becomes more embedded into research-driven academic institutions and medical research charities begin to strengthen their PPI activity.
